# Thirst and Drinking Paradigms: Evolution from Single Factor Effects to Brainwide Dynamic Networks

**DOI:** 10.3390/nu11122864

**Published:** 2019-11-22

**Authors:** Lawrence E. Armstrong, Stavros A. Kavouras

**Affiliations:** 1 Human Performance Laboratory and Department of Nutritional Sciences, University of Connecticut, Storrs, CT 06269-1110, USA; 2 Arizona State University, College of Health Solutions, Hydration Science Lab, Phoenix, AZ 85004, USA; stavros.kavouras@asu.edu

**Keywords:** dehydration, vasopressin, magnetic resonance imaging, neural network, motivation

## Abstract

The motivation to seek and consume water is an essential component of human fluid–electrolyte homeostasis, optimal function, and health. This review describes the evolution of concepts regarding thirst and drinking behavior, made possible by magnetic resonance imaging, animal models, and novel laboratory techniques. The earliest thirst paradigms focused on single factors such as dry mouth and loss of water from tissues. By the end of the 19th century, physiologists proposed a thirst center in the brain that was verified in animals 60 years later. During the early- and mid-1900s, the influences of gastric distention, neuroendocrine responses, circulatory properties (i.e., blood pressure, volume, concentration), and the distinct effects of intracellular dehydration and extracellular hypovolemia were recognized. The majority of these studies relied on animal models and laboratory methods such as microinjection or lesioning/oblation of specific brain loci. Following a quarter century (1994–2019) of human brain imaging, current research focuses on networks of networks, with thirst and satiety conceived as hemispheric waves of neuronal activations that traverse the brain in milliseconds. Novel technologies such as chemogenetics, optogenetics, and neuropixel microelectrode arrays reveal the dynamic complexity of human thirst, as well as the roles of motivation and learning in drinking behavior.

## 1. Introduction

**Figure 1:**
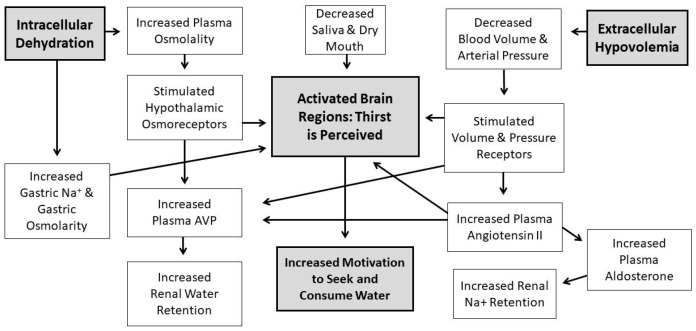
The thirst drive and motivation to seek/consume water are vital aspects of the homeostatic regulation of total body water volume and tonicity, in response to intracellular dehydration, increased plasma osmolality, decreased plasma volume, decreased blood pressure, and extracellular hypovolemia. Abbreviation: Na^+^, sodium.

**Figure 2:**
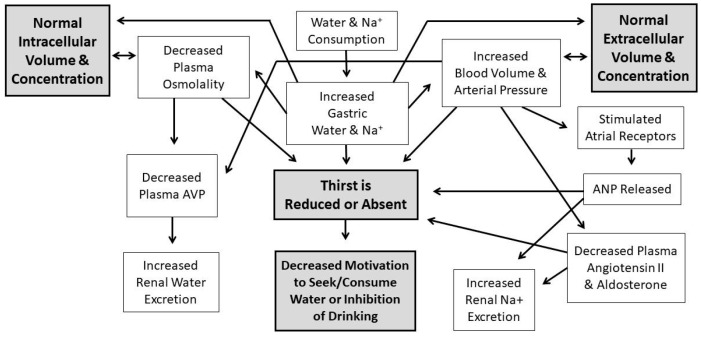
Homeostatic normalization of intracellular hydration, plasma osmolality, blood pressure, and extracellular volume (i.e., due to water and food intake), which result from a persistent, strong motivation to drink. These responses result in reduced thirst and decreased motivation to seek/consume water.

Water comprises over 80% of human brain, cardiac, skeletal muscle, kidney and gastrointestinal tissues [1]; it also is the medium in which metabolism, excretion, absorption, secretion and diffusion occur. Because water is essential for the survival of humans, selective pressures have forged mechanisms that regulate total body water (TBW) volume during periods of abundance, perturbation and insufficiency. When euhydrated, human TBW is regulated daily to within ±0.22% of body mass (±0.17 L) in a mild environment, and to within ±0.48% of body mass (± 0.38 L) during exercise-heat stress [2,3]. When assessed once per week, the average biological variability of TBW is 2.0% (0.9 L in a 74 kg healthy male) [4]. This vital stability, in a TBW pool of 44 L (i.e., 60% of 75 kg body mass), is accomplished via a complex, dynamic network of sensory nerves, brain integration, and neuroendocrine responses. Figure 1 and Figure 2 consolidate information from multiple publications [5,6,7,8,9,10,11] and represent the dynamic complexity of human fluid–electrolyte regulation. Figure 1 summarizes the varied homeostatic responses that occur (e.g., the sensation of thirst) in response to osmotically driven intracellular dehydration (left side) and extracellular hypovolemia (right side) that includes reduced circulating blood plasma and blood pressure. The latter state is a more immediate threat to life than cellular dehydration, thus interstitial fluid (i.e., part of the extracellular fluid between cells) serves as a buffer which can be mobilized as needed. When extracellular fluid volume depletion is extreme (e.g., >10% loss of body mass), physiological compensation includes vigorous drinking [12] and increased sodium consumption [13]. Figure 2 illustrates that a reduced motivation to seek and consume water (final state) ultimately results from normalization of intracellular hydration, body fluid osmolality, blood pressure, and extracellular volume.

## 2. Thirst and Drinking Behavior

The neuroendocrine aspects of TBW regulation are mostly autonomic and subconscious. Other than the clinical signs and symptoms which occur at severe levels of dehydration (e.g., headache, difficulty concentrating, or collapse [14]), thirst and the motivation to drink are among the few fluid-relevant sensations that humans perceive when mildly dehydrated, beginning at the level of 1%–2% body mass loss. As such, thirst is an integral, conscious aspect of TBW regulation and, during normal sedentary daily activities, is an adequate stimulus for total fluid replacement [15]. Although vitally important to optimal health and physiological functions, the definition, component parts, and mechanisms of thirst have evolved and have been debated since the 19th century [16,17], as presented in Table 1. The following paragraphs describe the ways that research findings, methodological/technological advances, and animal research have influenced these paradigm shifts.

Across intellectual disciplines, the definitions and perspectives of thirst are quite different. For example, the physiologist views thirst as a sensation induced by homeostatic imbalance (i.e., intracellular or extracellular water deficit, circulatory hypotension; Figure 1), which acts to counteract water deficiency [71]. Ironically, a few physiologists have published comprehensive reviews of water and electrolyte metabolism [95], regulation of cellular volume [96], and local renin–angiotensin systems mediating diverse physiological functions [97], with little or no mention of thirst and drinking. In contrast, social and behavioral psychologists often view thirst from an environmental perspective associated with meal times and cultural dietary norms, or factors such as motivation, incentive, and learning. This type of drinking ordinarily involves large inter-individual variability [98]. Human factors psychologists observe thirst from the perspectives of fluid availability, climate, and the sensory properties of fluids (i.e., temperature, palatability), with the goal of optimizing human performance, health and safety by designing equipment (e.g., water bottles), altering the environment (e.g., water availability), and modifying work tasks [99]. Cognitive neuroscientists observe activated brain regions, using the sophisticated imaging instruments described below, while test participants perform various tasks (e.g., drinking, swallowing, tasting). Finally, present-day chemogeneticists and optogeneticists study brain loci and communication pathways by observing chemically and genetically modified neurons in real time, to understand their roles in dehydration and thirst-motivated behaviors. Considering these specific content-area perspectives, the present review acknowledges that the thirst drive (i.e., measured via subjective rating scale [100,101]) is complex, dynamic, and distinct from drinking behavior—which is measured as the volume, concentration, temperature, palatability, and ingredients of fluid consumed [102,103].

**Figure 3:**
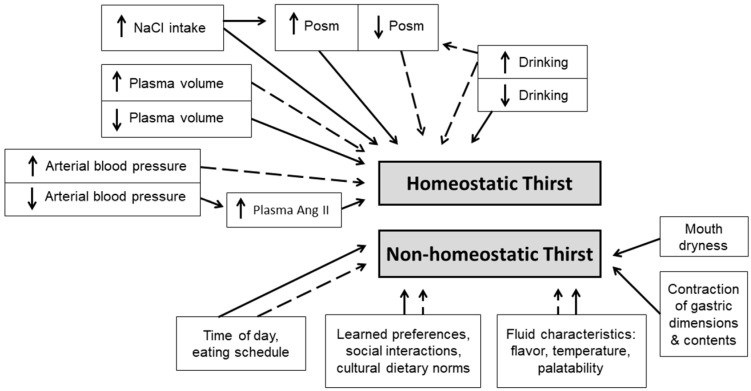
Factors that influence thirst are categorized as either homeostatic (i.e., volume, pressure, concentration) or relatively rapid non-homeostatic (anticipatory) inputs. Solid and dashed arrows represent, respectively, factors that increase and decrease thirst. Abbreviations: NaCl, sodium chloride; Posm, plasma osmolality; Ang II, angiotensin II.

Investigators have created models to facilitate understanding and communicate information about thirst and drinking behavior. One physiological paradigm [104] acknowledges that thirst arises from integration of physiological, psychological and environmental inputs to the brain (Figure 3). Physiological responses (Figure 1 and Figure 2) involving multiple organ systems maintain set points of osmolality, blood volume and blood pressure; this type of thirst has been named *homeostatic*. Published reviews of homeostatic thirst have presented a historical record of the advances resulting from both human and animal studies. These include the monograph published by A.V. Wolf [16] nearly 60 years ago, and the comprehensive review papers published by J.T. Fitzsimons. An initial Fitzsimons review [17] described early research (1817–1918), three classic theories of thirst, drinking behavior and satiety, the pharmacology of drinking, the rhythmicity of drinking and feeding schedules, disease-related dysfunctions of thirst, and the influences of electrical stimulation, intracellular dehydration, or extracellular dehydration on thirst. Subsequently, he focused on the stimuli that induce thirst in animals (e.g., dipsogens), as well as the hormonal and renal responses to thirst [6]. In 1998, Fitzsimons published a remarkable review of angiotensin, thirst and sodium appetite [7]; topics included renin- and angiotensin-induced drinking, the influences of peptides and neurotransmitters, and experimentally stimulated drinking. Even today, it is difficult to expand these classic, voluminous physiological publications. 

In contrast to homeostatic thirst, non-homeostatic thirst (Figure 3) is influenced by the taste and temperature of a fluid (i.e., alliesthesia [102,103]), mouth dryness, gastric distention, meal contents, meal timing [105], idiosyncratic learned, preferences, occupational schedules, social interactions, and cultural norms [75,98]. Both physiologists and cognitive neuroscientists have recognized that dehydrated humans drink to satiation rapidly across 3–10 min [74,106], causing decreased thirst and motivation to drink, well before elevated plasma osmolality and sodium return to normal concentrations (i.e., a process that may require 15–50 min [107,108,109], and before the consumed fluid is absorbed from the gut [110,111,112]. Thus, human homeostatic thirst is modulated by non-homeostatic oropharyngeal neural signals which rapidly reduce and limit total fluid intake in the presence of a persistent, strong motivation to drink [107]; these signals modulate satiety and oppose over-drinking [113].

## 3. Human Brain Imaging Technologies

Development of positron emission tomography (PET) imaging technology involved the noteworthy efforts of scientists, physicians and physicists who moved this technology from invention to research more than 30 years ago, then to clinical acceptance as an imaging modality during the years 1985–1995 [114]. During the initial years of development, PET scans were used almost exclusively to provide (a) an absolute measure of increased tissue metabolism (i.e., focusing on glucose utilization), especially in the diagnosis and progress of cancerous tumors [115,116], and (b) maps of human brain and heart function in three dimensions. Regional blood flow and metabolism also are determined via Fluorine-18-2-fluoro-2-deoxy-d-glucose and Oxygen-15-water PET imaging [116]. As a nuclear medicine technique, PET requires a cyclotron, specialized professional staff (e.g., a cyclotron engineer and nuclear medical technologist) and a positron-emitting radioisotope on a daily basis [117]. Today, new technologies allow noninvasive cell tracking via PET imaging of radiolabeled cells, with or without reporter genes. This technique involves three steps: stable expression of a reporter gene, ex vivo radiolabeling of co-incubated target cells, and transfer of cells for in vivo PET imaging [118].

Functional magnetic resonance imaging (fMRI) expanded enormously at the beginning of the 21st century and was dominated by basic neuroscience inquiry [119]. Published fMRI research spans a wide range of cognitive functions (e.g., taste, vision, hearing, memory, reward prediction, language, motor performance) and clinical disorders (e.g., multiple sclerosis, synesthesia, schizophrenia, psychopathology, post-traumatic stress disorder, migraine headaches, diabetes insipidus) [120]. Sequential fMRI images detect excess blood supply and the calculated oxygen delivery to a brain structure or region (i.e., a method that is named blood oxygen level-dependent responses, BOLD [119]); this method is an indirect measure of brain activity. As such, fMRI represents neural activations and metabolic activity, whereas its predecessor magnetic resonance imaging assessed only anatomical structures. In some research studies, fMRI is complimented by other measurements of brain physiology such as electroencephalography, transcranial magnetic stimulation, and near-infrared spectroscopy [120].

### 3.1. Imaging of Human Thirst and Drinking Behavior 

Both fMRI and PET imaging have advanced our understanding of the human brain regions that are activated during thirst (i.e., induced by intravenous injection, fluid restriction, or exercise); mouth irrigation, drinking, satiation; tongue protrusion, touching the tongue, swallowing; and taste stimuli. All of these processes are represented in Table 2, Table 3, Table 4 and Table 5. The ability to assess subjective sensations (i.e., using rating scales) during human imaging offers a great advantage over animal experiments, because the intensity of thirst, taste and other sensations can be correlated with changes of regional blood flow in the brain [92].

Table 2 summarizes the findings of 10 human laboratory studies in which investigators induced thirst. Because the experimental methods varied (i.e., thirst subsequent to intravenous hypertonic saline, fluid restriction, low-intensity exercise, and combinations of these methods), considerable differences exist in the brain regions that were activated. This suggests that the human brain processes different forms of dehydration (e.g., extracellular or intracellular) in unique ways, depending on the homeostatic disruptions of tonicity and volume experienced. This is reasonable, given that (a) the brain constantly monitors and regulates intravascular volume and plasma osmolality, and (b) fluid restriction and exercise-induced dehydration both decrease plasma volume but intravenous infusion expands plasma volume [124]. Table 2 also demonstrates that certain brain loci were activated in at least 50% of experiments, regardless of the method employed by investigators. These loci include the orbitofrontal cortex, frontal gyrus, inferior parietal lobe, temporal gyrus, cuneus, insular cortex (i.e., insula), anterior cingulate cortex, midcingulate cortex, and the thalamus. However, it is important to note that tongue movements, swallowing, and oropharyngeal sensations were not controlled, but concurrently activated specific brain loci during these experiments. 

Table 3 summarizes the same 10 human imaging studies presented in Table 2, but from the perspective of deactivation (i.e., determined on the basis of reduced PET and fMRI signal strength, below a pre-established threshold) of brain regions in response to mouth irrigation (n = 4) and subsequent oral drinking to satiation (n = 10). Investigators reported that insular cortex deactivations were observed in three out of four mouth irrigation experiments, but in no drinking-to-satiation trials. This may mean that the insular cortex integrates taste or temperature (i.e., from cool water) information but not satiety. In addition to these deactivated brain loci, it appears that the human brain responds to drinking and dilution of the extracellular fluid by activating inhibitory neural networks that generate an inhibitory emotion ([75]; see Figure 2); these inhibitory effects are described below.

Recognizing that the act of drinking to satiation, as presented in Table 3, involves neuromuscular and sensory actions, Table 4 presents the findings of investigations which focused on tongue protrusion, touching the tongue, and swallowing. Activations were observed in at least 50% of studies, in these eight brain loci: frontal gyrus, premotor cortex and primary motor cortex of the frontal lobe; somatosensory cortex and postcentral gyrus of the parietal lobe; insular cortex (observed in 73% of cited studies); temporal gyrus; and thalamus. As noted in Table 2 above, five of these loci were activated during dehydration and thirst; however, none of the publications summarized in Table 2 reported controlling for tongue movements, tongue sensations, or the act of swallowing. 

Table 5 summarizes the findings of five human studies that observed brain activations induced by applying solutions with distinct taste characteristics (e.g., salty, sweet, bitter) onto the tongue or into the mouth. The brain loci that were activated in at least 60% of studies included the orbitofrontal gyrus, frontal gyrus, and lingual gyrus of the frontal lobe, insular cortex, and the temporal gyrus. Of these, it is interesting that three were identified in both Table 2 (i.e., in response to dehydration and thirst) and Table 4 (i.e., in response to tongue movements, tongue touch, and swallowing). Thus, Table 5 suggests that taste is a sensory component of thirst. In support of this concept, both human and animal investigations have shown that ingestive appetite (i.e., fluids and food) is influenced by sensory characteristics [138], especially the desire to drink or not drink a fluid after it has been initially tasted [99,139]. 

Because little is known about human neural networks that produce the thirst sensation and motivate humans to drink, it is reasonable to ask, “What are the functions of activated brain loci in Table 2, Table 3, Table 4 and Table 5?” In response to this question, Table 6 presents a variety of evidence-based functions and sensations that have been associated with swallowing, thirst, taste, smell, and somatosensory information (e.g., temperature, touch, pressure, osmolality, stretch, movement). Although concurrently activated brain loci may represent neural circuits, this cannot be verified with fMRI and PET brain imaging methods. Human brain neural network algorithms have been developed [140] but have not been implemented in any of the studies presented in the present review. Table 6 also supports previous human fMRI studies which determined that multiple parallel interhemispheric neural pathways are involved in volitional swallowing [129,141], and are located in several spatially discrete cortical and subcortical loci, including a brainstem integrative network [126]. Volitional swallowing requires a complex sequence of carefully timed muscular contractions [132]. These events have been described in four stages: oral preparatory, oral transport, pharyngeal, and esophageal [127]. Numerous muscle actions and brain region activations are involved, as represented by the functions numbered 11–18 in Table 6 footnotes. In contrast, reflexive swallowing of water injected into the pharynx has been associated [130] only with the primary motor cortex (see function 17 in Table 6 footnotes). 

**Figure 4:**
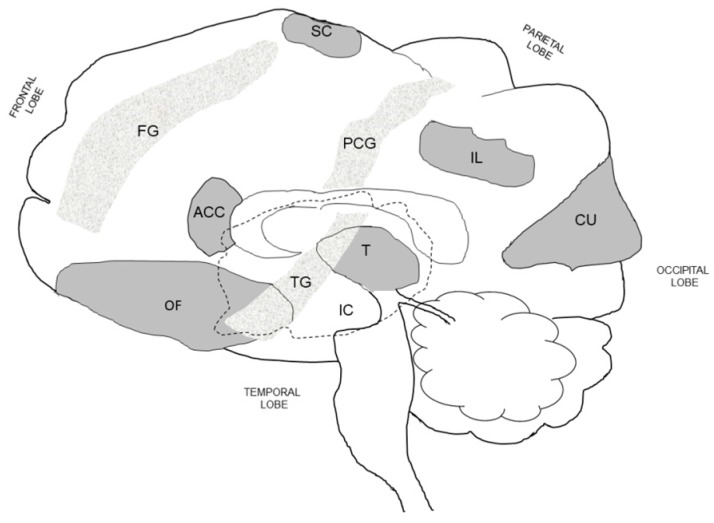
Idealistic drawing of human brain loci (dark shading) which have been associated with thirst, tongue movement, touching the tongue, swallowing, and taste (Table 2, Table 4 and Table 5). Three ridges (gyri) on the cerebral cortex surface are shaded lightly. The dashed region represents the insular cortex (IC) which lies deep within the lateral surface of the brain. Brain loci abbreviations are defined in the Table 2 footnote.

Figure 4 is an idealistic illustration drawn by the present authors, of the regions in the human brain that have been associated with thirst and drinking behavior, and based on the majority of brain imaging studies shown in Table 2, Table 4 and Table 5. Although our understanding of the neural pathways between these human brain loci is incomplete at present, it is widely accepted that some of these brain regions integrate a variety of sensory and motor inputs [129,137], and that complicated behavioral states (e.g., thirsty or satiated) are controlled by discrete clusters of neurons, some composed of only 1000–2000 individual cells in mice [143]. As examples, the following loci in Figure 4 are recognized as integrative hubs: orbitofrontal cortex (i.e., taste, small, visual, auditory, visceral inputs [137]), thalamus (i.e., mediates motivation and emotional drive [144]), insular cortex (i.e., processes sensory, emotions, movement, thirst, taste, touch inputs [129]), and anterior cingulate cortex (i.e., thirst, emotions, cognition, motor actions [42,145]).

### 3.2. Limitations of Human Brain Imaging 

Limitations exist in the conduct and interpretation of brain imaging studies. In Table 2, for example, the experimental protocols which induced thirst (i.e., some spanning >1 h) also altered intracellular and extracellular osmolality, water movements, as well as kidney, endocrine, and cardiovascular responses. Although these 10 studies (Table 2) focus on thirst, it is impossible to know the extent to which complex physiological responses (Figure 1 and Figure 2) influenced PET and fMRI findings. Similarly, Table 3 summarizes studies that focused on mouth irrigation and drinking to satiation, but the duration of these protocols ranged from 0.3 to 3.0 h. Within these studies, numerous digestive actions (e.g., peristalsis, swallowing) and orofacial movements (e.g., lips, tongue) may have occurred, introducing variability into the imaging data (Table 2 and Table 3). Further, with few exceptions [111], the investigators who studied thirst and drinking seldom distinguished or acknowledged the influence of subtle oropharyngeal movements, touch sensations (Table 4) or taste responses (Table 5) as coexisting variables. This is important because six brain regions that were activated in response to taste stimuli (Table 5; orbitofrontal gyrus, frontal gyrus, insular cortex, caudate nucleus, temporal gyrus, thalamus) also were activated in response to thirst (Table 2) and swallowing (Table 4). Due to this overlap, brain activation patterns by themselves do not allow unique identification of a task or the specific characteristics of a given task [146]. Further, some brain regions (e.g., anterior cingulate cortex, insula, thalamus) are highly integrative and serve as relay points for peripheral cardiovascular signals likely via midbrain nuclei. This makes it exceptionally difficult to delineate specific mechanisms for brain activations.

Human factors also may limit the interpretation of brain imaging data (Table 2, Table 3, Table 4 and Table 5) in three ways. First, inter-individual differences may be large, making it difficult to draw inferences about brain regions and networks. Large inter-individual variance has been acknowledged in studies of thirst and hunger [98], volitional swallowing [132], left versus right hemispheric dominance during swallowing [129], tongue movements [126], mental rotation, perception and memory [147,148]. Specifically, individual variance is due to differences in the ways that humans use, prioritize and integrate visceral, motivational, affective, and cognitive information; these differences arise from genetic influences, childhood developmental changes, depression, anxiety, eating disorders and subtypes of obesity [98]. Second, the selective attention which test participants devote to experimental interventions likely influence brain activations. A research group led by van Rinj [142] recorded fMRI scans of 27 women while they tasted water, fruit juice and tomato juice; additionally, these women were instructed to focus on different aspects of these fluids: pleasantness, taste intensity, and caloric contents. As a result of differential selective attention, brain region activations were different when women focused on the intensity and pleasantness of these fluids. This factor was not considered in the publications cited in Table 2, Table 3, Table 4 and Table 5; as such, the influence of selective attention is unknown and difficult to control during experiments. Third, the induction and satiation of thirst is difficult to replicate within a single session and across different days, mostly due to the many homeostatic and non-homeostatic factors [8,10,35,50,105] that influence thirst (Figure 1, Figure 2 and Figure 3) and the dynamic complexity of brain integration.

## 4. Neural Networks 

As described above, Table 1 illustrates the evolution of concepts regarding the nature and mechanism of thirst. Beginning with single factors (i.e., dry mouth, loss of water from tissues) in the 19th century, paradigms of thirst have progressed greatly due to investigations of brain loci and neural networks. However, neural networks provide four advantages over discrete brain loci, for the homeostatic regulation of body water and fluid osmolality, and initiation of thirst [149]. First, the organization of neural networks allows visceral somatosensory information to be broadly distributed from several relatively small loci which act as neural and endocrine ports of access into the brain. Second, the inherent adaptability, plasticity and information storage capability of neural networks are much greater than a single brain locus. Third, complimentary parallel neural networks provide redundancy so that damage or disease within a portion of the system is less likely to be catastrophic. Fourth, interconnected brain networks cooperate in three dimensions to accomplish functions efficiently [150]. 

## 5. Animal Research Compliments Human Brain Imaging

**Figure 5:**
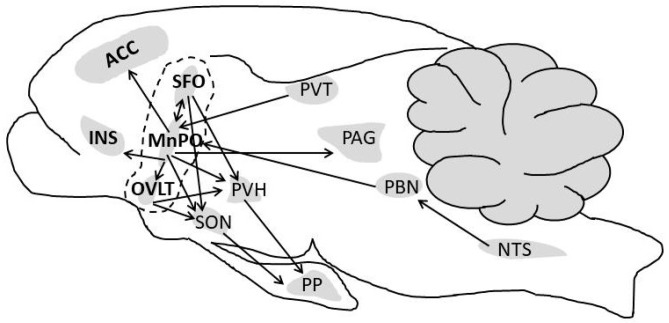
Rodent brain loci that have been associated (2016–2019) with dehydration-induced drinking [82,90,91,92,94,113,151]. In this idealistic illustration, arrows denote evidence-based neural circuits that integrate intracellular or extracellular signals and modulate thirst, drinking, rapid satiety, overdrinking, and aversive motivational drives. Abbreviations: IC, insular cortex (insula); SFO, subfornical organ; PVT, paraventricular thalamic nucleus; PP, posterior pituitary; additional brain loci are defined in the text and Table 2 footnote.

The rodent model of thirst and drinking behavior [63,93,151] has allowed identification of neurons that relay information regarding the status of plasma volume, vascular perfusion pressure, angiotensin II, ingested water passing through the mouth and throat, gastrointestinal water, extracellular sodium concentration and osmolality. Figure 5 illustrates and summarizes the findings of five rodent model publications which focused on thirst, thirst-related motivation, drinking behavior, and downstream signals to other brain loci. Although the authors of these investigations do not agree on all activated neural circuits and brain loci functions, they consistently agree that the lamina terminalis (a set of interconnected brain structures that coordinate the homeostatic responses to fluid imbalance; see dashed line in Figure 5) detects extracellular osmolality, angiotensin II, and fluid consumption [82,90,92,151]. The lamina terminalis amalgamates many input signals (e.g., plasma osmolality; examples appear in Figure 1, Figure 2 and Figure 3) and relays them to the anterior cingulate cortex and the insular cortex, to produce the conscious perception of thirst [92,124,151,152] and arginine vasopressin (AVP) release [93]. In addition, the thalamic paraventricular nucleus relays thirst-related signals to the median preoptic nucleus of the anterior hypothalamus (MnPO; a part of the lamina terminalis), as evidenced by the immediate, copious drinking that occurs when the paraventricular nucleus is photostimulated (see Section 5.1 below) [92]. Similarly, the nucleus of the solitary tract (NTS) and parabrachial nucleus (PBN) transmit anticipatory (non-homeostatic) oropharyngeal, baroreceptor, plasma sodium concentration, and upper gastrointestinal tract information via the vagus nerve to the MnPO [90,92]. As shown in Figure 5, the paraventricular nucleus of the hypothalamus (PVH) and the supraoptic nucleus (SON) are important downstream targets of the lamina terminalis that control release of AVP from the posterior pituitary into the circulation. Thus, PVH signaling influences not only urine production and blood pressure, but also the autonomic responses of heart rate and natriuresis [11].

Prior to the year 2000, the vast majority (91%) of the findings described in the preceding paragraph were discovered with the aid of two animal research methods: (a) lesioning/oblation of specific brain regions, or (b) microinjection/microinfusion. The former approach was utilized in more than 30 studies spanning 50 years, whereas the latter was employed in more than half of 86 publications (i.e., involving the awareness of thirst, drinking behavior, the functions of specific brain regions, and neural circuits) that were reviewed by the present author. The laboratory techniques of arterial or venous ligation, and electrical stimulation of specific brain loci, were employed in only 9% of these publications.

### 5.1. Optogenetics and Chemogenetics

Since the turn of this century, investigators have emphasized that it is not enough to know how individual brain loci and isolated networks function; studying how networks interact with each other is equally important. Today, the frontier is not brain network science, it is the science of *networks of networks* and the ways that networks rapidly connect and disconnect across the entire brain mass [153]. The development of two new laboratory techniques has uniquely enhanced our understanding of rodent brain regions and neural networks that influence thirst, motivation to seek water, and drinking (Table 1 and Table 7). The first is optogenetics, which allows researchers to visualize genetically targeted neurons in living animals, and to track electrical and biochemical events within living neural circuits [154]. Optogenetics utilizes light to control cells in living neurons that have been genetically modified to activate the membrane ion channels in light-sensitive proteins. This technology has given scientists the ability to stimulate or inhibit genetically defined populations of neurons with temporal resolution that is millisecond-precise [143]; in comparison, an fMRI brain scan requires several seconds to accomplish. These characteristics explain why optogenetics has been recognized as one of the most significant new methods of the present century, across all fields of science and engineering [155]. The second technique, named chemogenetics or pharmacogenetics, is similar to optogenetics but it utilizes chemically engineered molecules instead of light and light-sensitive membrane channels [156]. One form of chemogenetics employs directed molecular evolution. Known as Designer Receptors Exclusively Activated by Designer Drugs (DREADD), this tool is used by neuroscientists to activate specific neuronal circuits within the brain that influence thirst and body fluid homeostasis [143,157]. The combination of chemogenetics with imaging techniques in freely moving animals now makes it possible to analyze the complex whole-brain networks that are fundamental to behavior.

**Figure 6:**
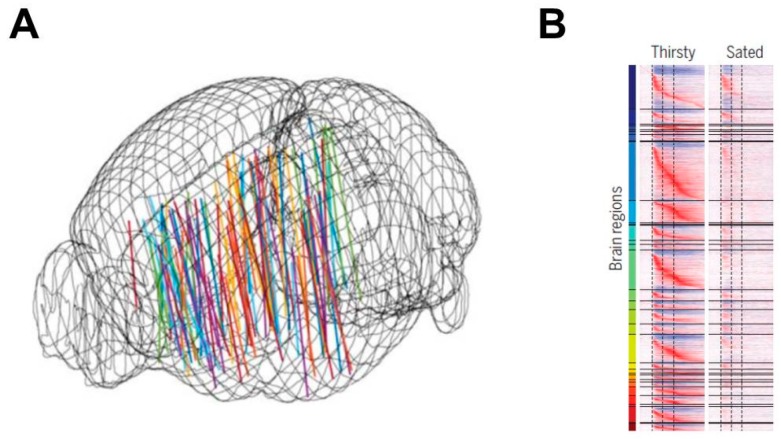
(**A**) Tracks of neuropixel electrode activations, recorded in mouse forebrain and midbrain regions during head-fixed, thirst-motivated choice behavior. (**B**) Brainwide activity dynamics of individual neurons from 31 brain loci, in response to an olfactory cue, while a mouse was both thirsty and sated. Reproduced with the permission of the publisher [94].

In 2019, W.E. Allen and colleagues [94] provided arguably the most enlightening thirst-related paradigm advance of the 21st century. Rather than utilizing fMRI scans of brain activations or neural circuits, this research team recorded high-density microelectrode array activations of approximately 24,000 individual neurons throughout 34 rodent brain loci, during several hundred thirst-related tasks (i.e., in a water restricted state, animals were trained to receive water as a reward for correct choices). Investigators specifically targeted thalamic and hypothalamic nuclei directly downstream of the MnPO, as well as diverse second-order regions (e.g., insular cortex) that were anatomically connected by axonal pathways to these downstream regions. As mice gradually consumed water, more than half of the recorded neurons responded to a water-predicting olfactory cue and sustained water acquisition activity. These procedures revealed a global representation of the thirst-motivational neural network, as depicted in Figure 6 [94]. Once satiated, this wave of brainwide activations (i.e., flow of information) was restricted to only a transient change of activity, with no motivated behavioral response. Subsequently, while mice were sated, localized optogenetic activation of hypothalamic thirst-sensing neurons rapidly converted brainwide activity to the pre-satiation state. The complexity of these brainwide neural network phenomena confirms research conducted more than a decade before. Bourque and colleagues [167] described the dynamic complexity of relationships among plasma osmolality, osmoreceptors, ingestive behaviors, sympathetic outflow, renal function, extracellular tonicity/volume, cardiovascular balance, and thermoregulation.

When compared to static brain imaging research (Table 2, Table 3, Table 4 and Table 5), the work of Allen et al. [94] is one of several rodent studies that exemplify the new behavioral insights and thirst-related paradigms which optogenetic and chemogenetic methods provide. For example, optogenetic or combined optogenetic–chemogenetic techniques have allowed the following discoveries to be made: (a) an innate brain circuit involving subfornical organ and organum vasculosum of the lamina terminalis neurons—from which signals are transferred downstream to the MnPO [84,91]—that can turn on and off an animal’s water-drinking behavior [80,82]; (b) thirst-promoting neurons (i.e., subfornical organ) respond to oropharyngeal inputs during eating and drinking as well as the composition of blood, to anticipate how food and water consumption will alter fluid balance in the future, and then adjust drinking behavior preemptively [93]; (c) thirst and salt appetite are driven by distinct groups of angiotensin II receptor neurons in the subfornical organ [85]. Far from the 19th century observations of thirst that involved single factors (Table 1), these techniques associate behaviors (i.e., motivation, incentive, seeking water or salt) with specific brain regions and neural circuits.

### 5.2. Limitations of Animal Models

The fluid–electrolyte balance of all vertebrates is subject to stringent homeostatic controls which maintain intracellular and extracellular ionic and osmotic conditions that are critical for normal cell functions [168]. Mammals, fish, amphibians, reptiles, birds, rodents, and humans share the common needs of maintaining osmolality, total body water, extracellular volume, and blood pressure. However, vertebrates obtain and conserve water and essential electrolytes via a wide range of taxonomic-specific evolutionary adaptations, including sodium appetite, restricted water loss from the body surface, regulation of urine contents, and water storage [60]. These vertebrate mechanisms of fluid–electrolyte balance are necessarily diverse, due to differences of environmental conditions (e.g., land, water, air, temperature, solar radiation, water availability) and life activities (e.g., avoiding predators, seeking food and water, migration) [60]. As a result, large species-specific differences of water consumption exist (i.e., expressed as % of body weight/24 h): man, 3%; dog, 5%; cattle, 6%, rabbit, 11%, and rat, 16% [3]. Even when comparing different strains of rats, divergent patterns of water intake have been reported during food deprivation (e.g., ranging from little change to an 80% decrease, relative to ad libitum baseline intake), prompting the authors to suggest that generalizations regarding rodent drinking behavior must be made with caution [169].

The human brain weighs 1.5 kg and consists of 86 billion neurons, whereas the mouse brain weighs 0.4 g and contains 70 million neurons [170]. Thus, it is relevant to ask, “Is it valid to generalize rodent thirst, drinking behavior, brain circuits, and neural networks to humans?” The answer to this question is not universally accepted; some research teams believe that neural and endocrine bases of thirst in rodents are similar to those of humans [9,42,92,93,124,171,172], whereas others emphasize rodent-human differences [124,172]. As noted above, the anterior cingulate cortex (ACC, acting in concert with the insular cortex) is essential to the conscious perception of thirst. However, the exact location, size, structure and connectivity of the ACC in nonhuman primates is not agreed upon by neurophysiologists, and it is not always obvious which areas of the rodent frontal cortex should be considered as equivalent to the human ACC [144]. Similar questions also arise in neuroscience fields other than thirst, where some researchers emphasize mouse–human anatomical and physiological similarities (e.g., Parkinson’s disease [173]; the neuroprotective benefits of exercise to counteract effects of aging [174]), some investigators acknowledge obvious mouse–human differences (e.g., size and complexity of the cerebral and cerebellar cortex, hemispheric dominance, hemispheric specialization [145,170,175], whereas others describe both similarities and differences in mouse and human brains (e.g., neural network organization in Alzheimer disease pathways [176]). Thus, after more than 125 years of experimental neuroscience, mouse and rat experiments may or may not have strong generalizability to humans, especially considering the fact that the mammalian cerebral cortex has proven to be far more variable across species than was believed two or three decades ago [175].

## 6. A Contemporary Public Health Problem: Low Daily Water Consumption

Public health surveys spanning decades have studies nutritional status and its association with health promotion and disease prevention; these surveys have revealed a remarkable variability in the 24 h total water intake (TWI) of apparently healthy individuals. For instance, in the third National Health and Nutrition Examination Survey, the lowest to highest decile of TWI was 1.69 to 7.93 L/24 h for men and 1.25 to 6.16 L/24 h for women [177]. Interestingly, the plasma osmolality of the lowest and highest water consumers were nearly identical for males (279 and 280 mOsm/kg) and females (277 and 277 mOsm/kg). In a cross-sectional study, investigators examined hydration biomarkers of healthy adults with low (LOW; 0.74 L/24 h) and high (HIGH; 2.70 L/24 h) water intakes [178]. They reported significantly elevated urinary hydration biomarkers (e.g., urine osmolality), plasma AVP, and plasma cortisol in LOW, without a LOW versus HIGH difference of plasma osmolality or thirst (i.e., this state has been described as underhydration [179]). In a subsequent intervention study involving women, Johnson and colleagues [180] evaluated the effects of water intake modification. At baseline, thirst and blood concentration were not different between groups but plasma AVP and urine osmolality were significantly elevated in LOW. Next, controlled water intake was increased in LOW and reduced in HIGH. Interestingly, serum osmolality was not different between groups at baseline, and did not change significantly during the 4 d water intervention for either group, giving the appearance of similar health states.

The principal public health question is whether chronically elevated plasma AVP (i.e., and concentrated urine) could increase the risk of chronic diseases, as previously had been described for kidney disorders. Multiple epidemiological studies from Sweden [181,182,183], United Kingdom [184], and France [185,186,187] have reported that elevated AVP, assessed by its surrogate marker copeptin, is positively associated with diabetes. In a study involving rats, AVP administration for four weeks induced glucose intolerance and hepatic steatosis [188]. Recently, a clinical trial observed 60 healthy males and females; osmotically elevated plasma AVP acutely impaired glucose regulation [189]. Similarly, several studies have linked LOW to the development of diabetes [185,186,187,190,191,192]. External to these effects on glucose regulation and diabetes, the pharmacologic blockade of AVP membrane receptors is currently under investigation in patients with rapidly progressing renal disease [186,187,188,189,190,191,192,193]. The above findings indicate that a low daily water intake activates homeostatic mechanisms involving AVP to maintain water and osmotic balance, and exposes LOW to an increased risk of chronic diseases. This raises important questions. Why are some people perfectly content to consume a very small TWI each day? Considering that only subtle physiological differences distinguish LOW from HIGH, what aspects of thirst are not homeostatic but rather involve trial and error, new information, or a strong dislike originating from a previous experience?

## 7. Learning, Motivation and Aversion

Thirst and drinking behavior are distinctly influenced by learning and motivation, in ways that classical physiologists have seldom considered or measured (Figure 1 and Figure 2 [162,163,166]) when they investigated homeostatic thirst. Table 7 describes several of these influences, as observed in both human and animal studies that span 165 years. The following are especially relevant: (a) drinking is behaviorally complex [160]; (b) hypovolemia elicits a true motivational state of thirst rather than mere reflexive drinking [56]; (c) the thirst drive and its related motivation to drink cause a heightened perceptual readiness to respond to environmental cues that may meet this need [164,166]; (d) specific human brain loci have been associated with perceptions, emotions, innate drives, memories, learning, and motor activities [88,157,165].

Although drinking is a fundamental behavior, the means by which the human brain transforms a need for water into a specific motivational drive is only superficially understood [84]. As a potential site of this transformation, the anterior cingulate cortex interests neuroscientists greatly. Heilbronner and Hayden [145] propose that ACC neurons (Figure 4 and Figure 5) link contexts with strategies by integrating diverse task-relevant information, to create a complex representation of the task environment and exert abstract control over decisions and actions. The ACC also has strong connectivity to motor centers [194]. These observations are consistent with the concept of a neural hub that integrates information from multiple homeostatic and non-homeostatic factors (Figure 3), then elicits thirst and motivates the host to seek and consume water [90,92,93,107,124,151].

A consistent and substantial body of evidence [152] describes the roles of the lamina terminalis, the ACC and the insular cortex (see Figure 4) as a neural pathway that generates thirst, motivation to seek water, and goal-directed drinking behavior. Homeostatic (i.e., plasma osmotic and endocrine) signals (Figure 3) are integrated by the lamina terminalis [124] and forwarded to the ACC, which translates these signals into emotional responses, arousal and affect by assigning cost–benefit values to behavioral options, considering alternative choices in an unpredictable or changing environment, and guiding decisions so that the most appropriate action is taken [152,195]. Anatomically, the ACC is uniquely located at the interface of the frontal cortex, the motor system and subcortical structures, allowing it to integrate multiple signals during development of motivation [195]. After attempting to acquire water (i.e., either successfully or unsuccessfully), the ACC purportedly updates host beliefs and internal models of decision making [196]. The insular cortex serves functions similar to those of the ACC [152,194] and also integrates neural signals from different loci in the lamina terminalis (e.g., subfornical organ, organum vasculosum of the lamina terminalis, median preoptic nucleus). Indeed, both the ACC and the IN are deactivated (i.e., fMRI signal strength) when drinking results in satiety; this state generates an aversive emotion that decreases motivation to drink and protects the host from over-drinking [123,194]. Thus, both the unpleasant effect of thirst and the pleasant effect of drinking regulate water consumption [82] and ultimately total body water balance (Figure 2).

Drinking behavior also is influenced by learning that involves past experiences, environmental characteristics, and pleasant or unpleasant sensations [139]. According to the classical “drive-reduction” hypothesis, animals learn specific behaviors that reduce the level of an aversive drive state (e.g., thirst). By reducing negative affect signals, a preference for cues associated with lessening of physiological need states (i.e., seeking and consuming water) can be learned [89]. It also is possible that humans learn to associate subtle cues (e.g., the time of day or a large sweat loss during prolonged exercise [197]) with impending dehydration and thus drink in anticipation of plasma volume and osmolality changes [13]. Interestingly, the ACC has been theoretically associated with learning in both rats and primates [145]. However, learning requires more than change in a single brain region; it requires multiple interconnected networks that reconfigure their connections during the learning process. Even when a person learns a relatively simple task, large portions of the brain become involved [198].

Two additional aspects of thirst and drinking behavior have been reported by investigators during the past five years. The first is based on the recognition that brain neurons within the MnPO encode an aversive motivational drive; these nerves represent a distinct homeostatic neuronal cell type with a distinct biological function that opposes thirst in mice [82,89]. The second involves fMRI evidence and extensive arguments regarding a human mechanism that opposes swallowing after rehydration and satiation of thirst. This inhibitory mechanism generates sensations of unpleasantness that are associated with the insular cortex, midcingulate cortex, amygdala, and periaqueductal gray matter. Previous research has implicated these regions in discomfort and aversion [75]. Subsequently, this research group conducted experiments involving ratings of swallowing effort and regional brain imaging [109], as participants prepared to swallow small volumes of liquid while they were thirsty and after they had overdrunk. After overdrinking, regional brain activations occurred in the motor cortex, prefrontal cortices, posterior parietal cortex, striatum, and thalamus when participants prepared to swallow. Effort ratings provided unequivocal support for swallowing inhibition, with a threefold increase in effort after overdrinking; these effort ratings were correlated with activations in the right prefrontal cortex and pontine regions of the brainstem. The authors concluded that swallowing inhibition in humans likely is a “hard-wired” process which helps the body to avoid the detrimental effects of overdrinking that could result in water intoxication and/or death [109]. Although not widely appreciated, both the aversive motivational drive and swallowing inhibition are part of the complex, dynamic regulation of fluid–electrolyte balance.

## 8. Summary and Implications for Future Research

The preceding paragraphs and Figure 3 emphasize that central nervous system homeostatic regulation of thirst and fluid intake integrates osmotic, ionic, hormonal, intracellular, and extracellular signals; concurrently, non-homeostatic regulation integrates oropharyngeal, environmental, social and cultural factors, fluid characteristics, and learned preferences. The motivation to seek and consume water arises from the integration and transfer of these signals to specific brain loci, and their conversion to decisions and actions via mechanisms that are not fully understood. Thus, thirst and drinking behavior have attracted, across more than 180 years (Table 1), the interest of investigators from numerous specialized research areas (e.g., physiology, medicine, behavioral psychology, cognitive neuroscience, optogenetics); these specialists measure different variables, use different methods and instruments, develop different concepts of thirst and drinking behavior, and as a general rule seldom communicate directly. Thus, most current paradigms and models are incomplete.

Most current concepts of thirst and drinking behavior have arisen from rodent studies, which may or may not be applicable to humans, and human brain imaging which has identified numerous active brain regions (e.g., concurrent with thirst or consuming water) but cannot causally ascribe specific motives or actions to each. Specifically, the following brain loci provide interesting promise (Table 1, Table 2 and Table 3) for future brain imaging studies of thirst and drinking: anterior cingulate cortex, insular cortex, orbitofrontal cortex, frontal gyrus, posterior cingulate cortex, thalamus, and cerebellum. In addition, Table 4 distinguishes the brain regions that apparently function in the muscular actions of swallowing or drinking (e.g., postcentral gyrus, primary motor cortex, premotor and supplementary motor cortex) but not in the awareness of thirst; importantly, the insular cortex was identified in 73% of Table 4 publications and in 100% of those cited in Table 5. However, considering only individual brain loci and localized neural pathways results in incomplete paradigms of thirst and drinking behavior. Today, the focus is on networks of networks, with thirst and satiety conceived in terms of hemispheric waves of neuronal activations [94] that move across the brain in milliseconds.

The future of thirst research lies in the newly developed techniques of chemogenetics, optogenetics, and neuropixel microelectrode arrays, which reveal important aspects of the dynamic complexity of human thirst, water seeking, and drinking. Although these recently developed invasive techniques (i.e., brain surgery, implanted electrodes, genetic manipulations) are limited to animal models and are not ethically possible in human research, it is our expectation that noninvasive methods someday will provide detailed paradigms that describe the conversion of numerous afferent signals into motivation, decisions, and actions that counteract perturbations of body water volume and concentration.

## Figures and Tables

**Table 1:** Evolution of concepts and biological techniques regarding the nature and mechanisms of thirst and drinking behavior.

Observations, Perspectives and Paradigms ^a^	Publications ^b^
When cholera patients were treated with intravenous saline and sodium bicarbonate, their intense thirst resolved.	[18]
Thirst is a local sensation and originates in the mouth and throat. Peripheral sensations (e.g., dry mouth, stomach contraction) caused by a water deficit become the instigating and sustaining conditions that motivate water seeking and drinking.	[19,20]
Thirst is a generalized sensation arising from the loss of water from tissues as dehydration progresses.	[21,22,23,24]
Water consumed orally, but not entering the stomach (i.e., exiting via esophageal fistula), does not stop the act of drinking. However, water injected directly into the stomach of an animal causes water intake to end.	[21]
Specific brain functions have precise anatomical localizations. Thirst arises from stimulation of a thirst center in the brain.	[25,26,27,28]
Anesthesia of the pharynx (i.e., bilateral section of nerves) does not affect thirst.	[22]
“True” thirst arises from lack of water in tissues whereas “false” thirst arises from dryness of mouth and throat. Moistening this region without restoring tissue water affords only temporary relief from thirst. A dry mouth is neither necessary nor sufficient to account for drinking in all circumstances.	[24]
Hypothalamic-pituitary neuroendocrine responses (unspecified) influence thirst.	[29,30,31]
Cellular dehydration stimulates thirst, with no change of cellular osmotic pressure.	[32,33]
Non-osmotic oropharyngeal sensations influence thirst and drinking behavior.	[34,35,36,37]
Electrical stimulation of specific brain areas induces drinking in unanesthetized animals, suggesting a localized thirst center.	[38,39,40,41,42]
Gastric distension satiates thirst.	[43,44]
Increasing the extracellular fluid (plasma) osmolality to varying levels allows determination of the plasma concentration at which thirst appears (i.e., the thirst threshold). The central drinking threshold decreases in response to intravenous administration of arginine vasopressin.	[45,46]
Hypothalamic damage alters thirst. Regulatory drinking is abolished by bilateral lesions in the lateral hypothalamus.	[47,48,49]
Rather than a single stimulus to drinking, multiple factors (osmotic pressure, sodium and chloride concentrations, intracellular and intracellular volumes) stimulate drinking.	[35,50]
Administration of active substances (i.e., saline, cholinergic agents) to specific brain loci via implanted brain cannulas induced drinking.	[41,51,52,53]
Hypovolemia is an independent and potent stimulus of thirst.	[33,54,55,56]
Cardiovascular feedback to the brain (i.e., volume, pressure, osmolality) modulates thirst.	[33,57,58,59,60,61,62,63]
A control model of thirst was developed on the basis of physiological research and was simulated using a digital computer.	[64,65]
The renin-angiotensin system mediates thirst and stimulates a search for water. Angiotensin II (Ang II) stimulates drinking. Injection of an Ang II antagonist (saralasin) directly into cerebral spinal fluid blocks drinking. Ang II also drives salt appetite and consumption of salt.	[10,61,66,67,68,69,70]
Loss of intracellular water and extracellular water stimulate drinking independently and, when loss of body water affects both, the signals to drink are additive. This phenomenon is named the “double depletion hypothesis of thirst”.	[71,72,73]
The kidneys may influence drinking behavior by affecting the: (1) volume of water in the body (e.g., a diuretic may cause thirst secondary to urinary water loss without influencing thirst directly); (2) amount of solute in the body (e.g., while leisurely consuming a hypertonic beverage, some of the salt is excreted); and (3) release of angiotensin II, which stimulates drinking at specific brain loci (i.e., subfornical organ, organum vasculosum of lamina terminalis).	[6]
Two thirst states exist. The first is induced by a state of physiological need (i.e., reestablishes homeostasis of volume and concentration after dysequilibrium occurs; it is regulatory), and the second is not primarily regulatory (i.e., non-homeostatic).	[7]
Multiple variables stimulate thirst and drinking behavior of rats: intragastric sodium chloride, intragastric water, increased or decreased arterial blood pressure, decreased plasma volume, increased or decreased plasma osmolality, and increased plasma or local angiotensin II concentration.	[8]
The anterior cingulate cortex is recognized as a thirst center in the brain and is associated with consciousness of thirst and the pleasantness of drinking.	[74,75]
Utilizing optogenetic laboratory techniques, neuroscientists can elucidate neuron dynamics during thirst and drinking behavior, as well as the downsteam pathways by which neurons transmit information to other brain regions. See text for methodological details. ^c^	[76,77,78]
Optogenetic techniques have allowed identification of specific rodent brain loci that (a) establish a persistent aversive state when the animal is dehydrated; (b) regulate motivation for water intake, thirst, and their circadian influences; (c) induce and reduce water consumption; (d) distinguish selective water and salt intake; and (e) detect water at the tongue via taste sensations.	[79,80,81,82,83,84,85,86,87]
Utilizing chemogenetic laboratory techniques, neuroscientists have discovered relationships between brain activity, brain neural circuits, thirst, and drinking behavior in freely moving animals. See text for methodological details. ^c^	[88]
Combining chemogenetic and optogenetic methods, investigators have identified rodent brain loci that (a) induce drinking while consuming food (i.e., prandial drinking), (b) suppress food intake when water is unavailable (dehydration anorexia), (c) stimulate thirst-quenching signals that lead to rapid satiety following consumption of fluids but not solids, (d) are influenced by circulating hormones (i.e., Ang II), and (e) interpret environmental cues associated with water intake.	[89,90,91,92]
Two distinct neural populations in the brain trigger or suppress thirst. This suggests an innate brain circuit that initiates and stops animal water-drinking behavior, and likely functions as a center for thirst control in the brains of mammals.	[80]
Thirst-promoting neurons in the brain respond to inputs from the oral cavity during eating and drinking, which they then integrate with information about blood composition. This suggests a neural mechanism to explain behaviors such as the prevalence of drinking during meals, the rapid satiation of thirst, and the thirst-quenching influence of oral cooling.	[93]
The aversive quality of thirst, and the motivation to drink, drive the desire to quench thirst. Localized brain neuron activity is proportional to the strength of this aversive emotion (i.e., negative valence).	[82,83,93]
Sensory neurons that perceive extracellular osmolality, volume and blood perfusion pressure (thereby producing the sensation of thirst) converge on the same brain region as the neurons that release arginine vasopressin (AVP, antidiuretic hormone). As such, elevated extracellular fluid osmolality stimulates the sensation of thirst to promote water intake, and the release of vasopressin that enhances water reabsorption in the kidney.	[63]
Several hormones associated with eating and satiety have been proposed to modulate thirst neurons and vasopressin release; these include amylin, cholecystokinin, ghrelin, histamines, insulin, and leptin.	[63]
The water and salt content of the gastrointestinal tract are precisely measured and communicated to the brain, to control the drinking behavior of mice. This osmosensory signal (a) involves the vagus nerve, (b) is integrated with oropharyngeal and blood-borne signals, and (c) is transmitted from the gut to forebrain neurons that control thirst and vasopressin release.	[11]
In the rodent brain, activation of approximately 24,000 neurons in 34 brain loci revealed a global brainwide representation of a thirst-motivated state. This state appears to moderate the propagation of sensory information and its transformation into behavioral output.	[94]

^a^ most of the above observations, perspectives, and paradigms arise from research involving rodents; ^b^ publications prior to 1920 are reviewed thoroughly by Fitzsimons (1973); ^c^ see Section 5.1 (below) Optogenetics and Chemogenetics.

**Table 2:** Activation (A) of human brain regions and loci in response to experimentally induced thirst.

**Method of Inducing Thirst**	**Frontal Lobe**			**Parietal Lobe**				**Temporal Lobe**		**Occipital Lobe**			**Insular Cortex**		**Striatum**		**References**
	**OF**	**FG**	**MC**	**IL**	**PCG**	**SC**		**TG**		**LG**		**CU**			**PT**	**CN**	
T_IV_	A	A			A			A								A	[110] ^a^
T_IV_	A	A			A	A		A		A		A	A			A	[74] ^a^
T_IV_										A		A					[111] ^a,b^
T_IV_		A			A			A		A		A	A			A	[108] ^a,c^
T_IV_		A	A	A				A		A		A	A				[121] ^a^
T_IV_	A	A	A	A		A		A				A					[112] ^a^
T_FR_	A			A									A				[122] ^c^
T_FR_								A					A				[123] ^c^
T_30_	A	A		A				A							A		[124] ^d^
T_60_	A	A		A				A				A	A		A		[75] ^c^
**Method of Inducing Thirst**	**Cingulate Cortex**			**Limbic System**						**Brain Stem**		**Cerebellum ^e^**					
	**ACC**	**MCC**	**PCC**	**HI**	**PG**	**T**	**HY**	**LT**	**A**	**P**	**PAG**	**I–IV**	**V**	**VI–VII**	**VIII–IX**	**DN**	
T_IV_	A			A	A	A					A		A	A			[110] ^a^
T_IV_	A	A	A	A	A	A			A	A							[74] ^a^
T_IV_		A	A		A	A						A	A	A	A		[111] ^a,b^
T_IV_	A		A		A	A	A	A		A		A	A	A	A		[108] ^a,c^
T_IV_	A	A															[121] ^a^
T_IV_	A	A				A											[112] ^a^
T_FR_	A										A						[122] ^c^
T_FR_									A								[123] ^c^
T_30_	A		A					A									[124] ^d^
T_60_	A	A				A				A	A	A	A	A	A	A	[75] ^c^

^a^ positron emission tomography; ^b^ magnetic resonance imaging; ^c^ functional magnetic resonance imaging; ^d^ fMRI with pulsed arterial spin labeling; ^e^ I–IV, anterior hemisphere, lingula, central; V, anterior quadrangulate lobule, culmen; VI–VII, posterior quadrangulate lobule, declive, superior semilunar lobule; VIII–X, pyramis, uvula, nodulus, biventral lobule, tonsillar; DN, dentate nucleus. Abbreviations: T_IV_, hyperosmolar thirst induced via hypertonic saline; T_FR_, thirst induced by 6-8 h fluid restriction; T_30_, thirst induced by 30 min of cycling exercise plus 4.5 h fluid restriction; T_60_, thirst induced by 60 min of cycling exercise with no fluid intake; OF, orbitofrontal cortex; FG, frontal gyrus; MC, primary motor cortex; TG temporal gyrus; IL, inferior parietal lobule; PCG, postcentral gyrus; SC, somatosensory cortex; TG, temporal gyrus; LG, lingual gyrus; CU, cuneus; PT, putamen; CN, caudate nucleus; ACC, anterior cingulate cortex; MCC, midcingulate cortex; PCC, posterior cingulate cortex; HI, hippocampus; PG, parahippocampal gyrus; T, thalamus; HY, hypothalamus including the organum vasculosum of the lamina terminalis (OVLT); LT, lamina terminalis; A, amygdala; P, pons; PAG periaqueductal gray matter.

**Table 3:** Deactivation (D) of human brain regions in response to mouth irrigation and drinking to satiation.

**Deactivation Stimulus**	**Frontal Lobe**			**Parietal Lobe**				**Temporal Lobe**		**Occipital Lobe**		**Insular Cortex**	**Striatum**			**References**
	**OF**	**FG**	**MC**	**SL**	**IL**		**PCG**	**TG**		**LG**	**CU**		**PT**		**CN**	
MI, S	D	D						D							D	[110] ^a^
MI, S												D				[74] ^a^
MI, S	D											D				[122] ^b^
MI, S		D		D						D	D	D				[123] ^b^
S																[111] ^a^
S		D													D	[108] ^a,b^
S																[121] ^a^
S	D	D														[112] ^a^
S																[124] ^c^
S																[75] ^b,d^
**Deactivation Stimulus**	**Cingulate Cortex**			**Limbic System**						**Brain Stem**		**Cerebellum**				
	**ACC**	**MCC**	**PCC**	**HI**	**PG**	**T**	**HY**	**LT**	**A**	**P**	**PAG**	**I–IV**	**V**	**VI–VII**	**VIII–X**	
MI, S				D	D	D										[110] ^a^
MI, S	D	D	D		D	D			D							[74] ^a^
MI, S											D					[122] ^b^
MI, S		D														[123] ^b^
S												D	D	D	D	[111] ^a^
S	D		D							D					D	[108] ^a,b^
S		D														[121] ^a^
S		D				D										[112] ^a^
S	D															[124] ^c^
S																[75] ^b,d^

^a^ positron emission tomography; ^b^ functional magnetic resonance imaging; ^c^ fMRI with pulsed arterial spin; ^d^ no deactivations were considered or reported. Abbreviations: MI, mouth irrigation; S, drinking to satiation; D, reduced brain image signal strength, below a predetermined threshold; SL, superior parietal lobule; additional brain loci abbreviations appear in Table 2 footnote.

**Table 4:** Human brain region activations (A) in response to tongue movements, touching the tongue, water held in the mouth, and swallowing.

**Experimental Condition**	**Frontal Lobe**					**Parietal Lobe**				**Occipital Lobe**				**Insular Cortex**	**Striatum**		**References**
	**OF**	**FG**	**MC**	**RA**	**PMC**	**PN**	**IL**	**PCG**	**SC**	**CU**	**OO**	**LG**	**VC**		**PT**	**CN**	
T					A			A	A								[125] ^a,b^
TE		A			A	A		A	A						A		[126] ^d^
TE	A	A	A		A	A	A		A	A				A			[127] ^d^
VS							A	A	A					A	A		[128] ^a,b^
VS		A	A					A	A					A			[129] ^d^
VS		A	A		A	A		A	A	A	A			A			[126] ^d^
VS, R			A		A				A				A				[130] ^d^
W	A	A					A	A	A					A		A	[131] ^a,b^
SW	A				A			A						A			[132] ^a,b,c^
SW		A	A	A	A			A	A			A		A			[131] ^a,b^
SW		A	A		A	A	A	A	A	A				A			[127] ^d^
**Experimental Condition**	**Temporal Lobe**				**Cingulate Cortex**			**Limbic System**		**Brain Stem**			**Cerebellum**				**References**
	**TG**	**FG**		**AA**	**ACC**	**PCC**		**T**	**A**	**DB**	**P**	**MB**	**I–IV**	**V**	**VI–VII**	**VIII–X**
T	A																[125] ^a,b^
TE					A			A									[126] ^d^
TE	A							A		A		A					[127] ^d^
VS	A	A			A			A							A	A	[128] ^a,b^
VS																	[129] ^d^
VS	A				A												[126] ^d^
VS, R						A											[130] ^d^
W	A							A			A			A	A	A	[131] ^a,b^
SW				A	A				A	A					A		[132] ^a,b,c^
SW												A		A	A	A	[131] ^a,b^
SW								A				A		A		A	[127] ^d^

^a^ positron emission tomography; ^b^ magnetic resonance imaging; ^c^ transcranial magnetic stimulation mapping; ^d^ functional magnetic resonance imaging. Abbreviations: T, voluntary tongue protrusion and bilateral touching the tongue; TE, voluntary tongue elevation inside the mouth; VS, voluntarily swallowing own saliva on cue, no injection; R, reflexive swallowing induced by injecting water into the pharynx; W, voluntarily held injected deionized water in mouth; SW, voluntarily swallowed injected deionized water on cue; RA, Rolandic area; PMC, premotor and supplementary motor cortex; PN, precuneus; OO, occipital operculum; VC, visual cortex; FG, fusiform gyrus; AA, auditory association cortex; DB, dorsal brainstem; MB, midbrain; additional brain appear loci abbreviations in footnotes of tables above.

**Table 5:** Activation (A) of human brain regions and loci in response to taste stimuli.

**Taste Stimuli**	**Application Method**	**Frontal Lobe**			**Parietal Lobe**					**Occipital Lobe**		**Insular Cortex**	**Striatum**	**References**
		**OF**	**FG**	**LG**	**IL**	**PCG**	**SC**	**SG**	**AG**	**LG**			**CN**	
D, Na	I											A	A	[133] ^a^
D, F	P	A	A	A						A		A		[134] ^a,b^
Na, Sac	I	A		A						A		A	A	[135] ^b,d^
F	S		A	A			A	A	A			A		[136] ^c^
F	I	A	A									A		[137] ^c^
**Taste Stimuli**	**Temporal Lobe**	**Cingulate Cortex**			**Limbic System**			**Brain Stem**		**Cerebellum**				**Reference**
	**TG**	**ACC**		**MCC**	**HI**	**PG**	**T**	**P**		**I–IV**	**V**	**VI–VII**	**VIII–X**	
D, Na	A	A			A	A	A							[133] ^a^
D, F														[134] ^a,b^
Na, Sac	A			A	A	A	A							[135] ^b,d^
F	A													[136] ^c^
F		A												[137] ^c^

^a^ positron emission tomography; ^b^ magnetic resonance imaging; ^c^ functional magnetic resonance imaging; ^d^ magnetoencephalography. Abbreviations: D, deionized water; Na, sodium chloride solution; I, injected into mouth via tube; F, 4–8 different taste stimuli were applied to the tongue; P, filter paper placed on tongue; Sac, saccharine solution; S, sip and spit protocol; SG, supramarginal gyrus; AG, angular gyrus; additional brain loci abbreviations are defined in previous table footnotes.

**Table 6:** Proposed functions of human neural networks and concurrently activated brain regions and loci. ^a,b.^

**Frontal Lobe**					**Parietal Lobe**					**Temporal Lobe**	**Occipital Lobe**			**Insular Cortex**	**Striatum**			**References**
**RA**	**OF**	**FG**	**MC**	**PMC**	**PN**	**SG**	**IL**	**PCG**	**SC**	**TG**	**OO**	**LG**	**CU**		**PT**	**CN**		
									1,4					1,4				[121]
									1,4					1,4				[112]
	4	1,4			1					1,4				1		4		[124]
		1,4												1,4				[108]
	2	2												2				[122]
	5													6				[75]
	2														11,12	11,12		[110]
	4													4				[74]
														5				[123]
5,10	5,10	5,10												5,10	5,10			[142]
	7,9a	7										9a						[134]
										8		8		7,8		8		[133]
	9a	9a												9a				[137]
			13		15	15		15	19				15	15				[126]
								15		15				15	15			[128]
				15					15	15				15				[132]
	15		15						15						15	15		[109]
		16							15					16				[129]
16											16			16				[131]
		15	17	15	15								15	15				[130]
		18		15,18	18		15,18	15,18	15,18	18			18	15,18	18			[127]
**Cingulate Cortex**					**Limbic System**							**Brain Stem**		**Cerebellum**				
**ACC**	**ACG**	**MCC**		**PCC**	**HI**	**PG**	**T**	**HY**	**LT**	**A**	**MB**	**P**	**PAG**	**I–IV**	**V**	**VI–VII**	**VIII–IX**	
1,4				1,4		1,4	1,4	1,4							1,4	1,4		[121]
							1,4	1,4						1,4	1,4	1,4	1,4	[112]
1		1,4		1,4		1,4	1,4		1							4	4	[124]
	1,4						1,4							1,4	1,4	1,4	1,4	[108]
																		[122]
		6		5						6	6		6					[75]
2,11,12									2			12	12					[110]
3,4,14				3,4,14	4,14	4	3,4,14	3		3								[74]
5																		[123]
5,10							5,10			5,10								[142]
	9a							9a		7,9a								[134]
	8					8	8											[133]
9a																		[137]
13																		[126]
							15									15	15	[128]
										15						15		[132]
							15											[109]
																		[129]
																		[131]
15																		[130]
15,18							15,18				15,18				15	15		[127]

^a^ these evidence-based functions are described in the Introduction and Discussion sections of each publication; ^b^ concurrently-activated brain regions and neural networks are suggested by identical numbers within each study (row). Fluid homeostatic functions: 1, responds to changes of plasma osmolality and/or sodium concentration. Non-homeostatic thirst, taste, and sensory functions: 2, responds to water intake or mouth irrigation; 3, dry mouth sensation; 4, thirst sensation; 5, discriminates fluid pleasantness/unpleasantness; 6, discriminates fluid pleasantness/unpleasantness during overdrinking when satiated; 7, integrates taste and olfactory sensations, recognized as flavor; 8, taste sensation specific to salt; 9a, integrates multiple taste stimuli; 9b, integrates multiple thirst-related stimuli; 10, processes the intensity of fluid characteristics. Non-homeostatic tongue movement, swallowing, and facial functions: 11, muscular motor activity; 12, coordinates motor outputs related to appetitive reward (e.g., thirst); 13, plans and executes movements; 14, processes spatial memory and visual association; 15, regulates volitional swallowing; 16, processes somatosensory or motor information during repetitive swallowing; 17, regulates reflexive swallowing; 18, integrates voluntary tongue elevation inside the mouth; 19, facial sensations. Abbreviations: ACG, anterior cingulate gyrus; other brain loci are defined in the text and Table 2 footnote.

**Table 7:** The importance of incentive, motivation and learning in thirst and the drinking behavior of rodents, primates, and humans.

Paradigms	Authors
Peripheral sensations (e.g., dry mouth, stomach contraction) caused by a water deficit become the instigating and sustaining conditions that motivate water seeking and drinking.	[19,20]
We will not fully understand thirst until we acknowledge motivation and the elusive neurological questions it poses: how does the urge to drink arise in the brain, what compels animals to anticipate water consumption, what creates the hedonic state of thirst, and what gives rise to the excitement of water need or the pleasure of consumption?	[72]
Animals can learn to drink in specific situations via classical conditioning. For example, neutral stimuli (i.e., having no effect on behavior) elicit drinking following their repeated association with thirst-inducing treatments (e.g., water deprivation, hypovolemia, hypertonic saline injections). Conditioned physiological changes do not elicit this drinking behavior. ^a^	[158]
Animals can learn to associate specific tastes with the effects that result after fluid consumption; this learning influences subsequent experiences with those fluids. ^a^	[159]
Palatability of a fluid (i.e., pleasant flavor, aversive taste, temperature) can override the homeostatic control (i.e., plasma hyperosmolality, extracellular volume depletion) of fluid balance in humans.	[138]
Small animal drinking is behaviorally complex. A small animal drinks in connection with eating, in anticipation of thirst, and because it learns the location of a water source. It paces drinking across a diurnal cycle. It will forego drinking if too much searching is required. ^a^	[160]
Motivation of animals can be determined by measuring: (a) how hard they work (e.g., number of times they will press a lever, distance they will travel in a maze) to obtain water, rather than how much water is consumed before satiety mechanisms terminate drinking; and (b) the extent to which they will tolerate aversive consequences (e.g., water containing different concentrations of bitter tasting quinine, electrical shock) in order to obtain water. ^b^	[104]
Motivation-specific response systems exist in the primate brain. That is, some neurons respond to the sight or taste of food but not water, and other neurons respond to the sight and/or taste of water but not food. Such motivational specificity is required of a system that guides and controls hunger- or thirst-motivated behavior.	[161]
Behavioral responses depend partly on physiology and vice versa. In some situations, normal drinking in rats may be largely due to non-physiological factors.	[162]
The arousal of a motive has no necessary connection with homeostatic regulation. Some motives arise without needs, and some needs arise without motives.	[163]
Much physiology-induced human behavior is learned. Virtually all ingestive appetite is acquired and is influenced by sensory characteristics (i.e., taste, smell, texture) or the cooling (i.e., pleasurable) effects of water. This is true for babies, young children and adults.	[139]
The initiation or termination of drinking could result from past personal experiences. A variety of associations with the consequences or outcomes of drinking in specific situations may leave a desire (i.e., to subsequently drink or not to drink certain beverages), when those situations are next encountered.	[139]
Basic needs and motives such as thirst cause a heightened perceptual readiness to environmental cues that are instrumental in satisfying these needs (e.g., recognizing a water source that otherwise might go unnoticed).	[164]
Utilizing chemogenetic and optogenetic laboratory techniques, neuroscientists activate specific brain neurons to identify the circuitry and cellular signals that influence/generate behaviors, innate drives, memories, learning, and motor functions. See text for methodological details. ^c^	[88,151,157,165]
The theory of incentive motivation states that the power of external stimuli (i.e., visual recognition) is calibrated dynamically, based on the current body state of the organism (e.g., motivation is high when the body is dehydrated and low when normally hydrated).	[166]
The activity of dehydration-activated neurons (i.e., in specific brain regions) establishes a scalable, persistent, and aversive internal state that dynamically controls thirst-motivated behavior. This state motivates drinking to quench thirst. Localized brain neuron activity is proportional to the strength of this aversive emotion (i.e., negative valence). ^a^	[79,81,82,83,93]
Rodent brain loci have been identified that control motivational processes such as water seeking, drinking, and cessation of drinking.	[90,92]
The motivated behavior of rodents to seek and consume water is regulated by thirst; this regulation involves modulation of brainwide neural population dynamics. ^c^	[94]

^a^ researchers cannot know if an animal is thirsty; when referring to non-humans, the acts of seeking and consuming water are assumed to indicate that a thirst-like drive exists; ^b^ these measurements will not always correlate with the amount of water consumed during free access (*ad libitum* drinking) because the amount consumed reflects the amount of water required to terminate drinking, rather than the initial motivation of an animal to obtain water; ^c^ see section (above) 5.1 Optogenetics and Chemogenetics.
